# Retroperitoneal Hemorrhage: A Rare Cause of Severe Acute Maternal Morbidity and Perinatal Mortality

**DOI:** 10.7759/cureus.69057

**Published:** 2024-09-10

**Authors:** Rimpi Singla, Shalini Venkatappa, Sreedhara B Chaluvashetty, Girdhar S Bora, Rashmi Bagga

**Affiliations:** 1 Obstetrics and Gynecology, Postgraduate Institute of Medical Education and Research, Chandigarh, IND; 2 Radiodiagnosis and Imaging, Postgraduate Institute of Medical Education and Research, Chandigarh, IND; 3 Urology, Postgraduate Institute of Medical Education and Research, Chandigarh, IND

**Keywords:** angiomyolipoma, nephrectomy, pregnancy, retroperitoneal hemorrhage, spontaneous adrenal hemorrhage

## Abstract

Retroperitoneal hemorrhage in the form of spontaneous adrenal hemorrhage or bleeding into a renal tumor can have varied and non-specific presentations in pregnancy. In the absence of risk factors, these life-threatening conditions are rarely suspected. We present our experience with three patients who presented to us in the third trimester of pregnancy with hemorrhage in retroperitoneal organs. One of the patients had a spontaneous adrenal hemorrhage and the other two had a hemorrhage in the renal tumor. None of the patients was known to have pre-existing tumors, coagulopathy, or trauma. Both the patients with hemorrhage in the renal tumor had intrauterine fetal demise at the time of presentation. Immediate resuscitation and recruitment of a multidisciplinary team resulted in optimal maternal outcomes in all cases and a healthy fetal outcome in the patient with adrenal hemorrhage.

## Introduction

Retroperitoneal hemorrhage of renal or adrenal origin can rarely become the cause of catastrophic outcomes in an uncomplicated pregnancy [1]. Risk factors include tumor, trauma, sepsis, coagulopathy, pre-eclampsia, postpartum hemorrhage, and antiphospholipid antibody syndrome [1]. Spontaneous adrenal hemorrhage during pregnancy is a rare event despite physiological hyperplasia, hypertrophy, and rich blood supply of the adrenal cortex. Even in the presence of risk factors, it is not suspected due to rarity and non-specific presentation [1,2] that varies from an incidental finding to shock or adrenal failure [3,4].

Renal angiomyolipomas are benign tumors of the kidney that are mostly asymptomatic and have a slow and consistent growth rate [5]. During pregnancy, these tumors may be complicated by hemorrhage [6,7]. Hence, some studies have recommended elective treatment in the form of selective arterial embolization (SAE) and nephron-sparing surgery or nephrectomy in women of childbearing age [8].

In the absence of knowledge of pre-existing lesions, the diagnosis of adrenal or renal hemorrhage may be delayed as pregnant women would present to an obstetrician for any emergency and not to the urologist. We report our experience with three such patients in the third trimester of pregnancy with a focus on when to suspect, how to diagnose, and the need to individualize the treatment.

## Case presentation

Case 1

**Figure 1:**
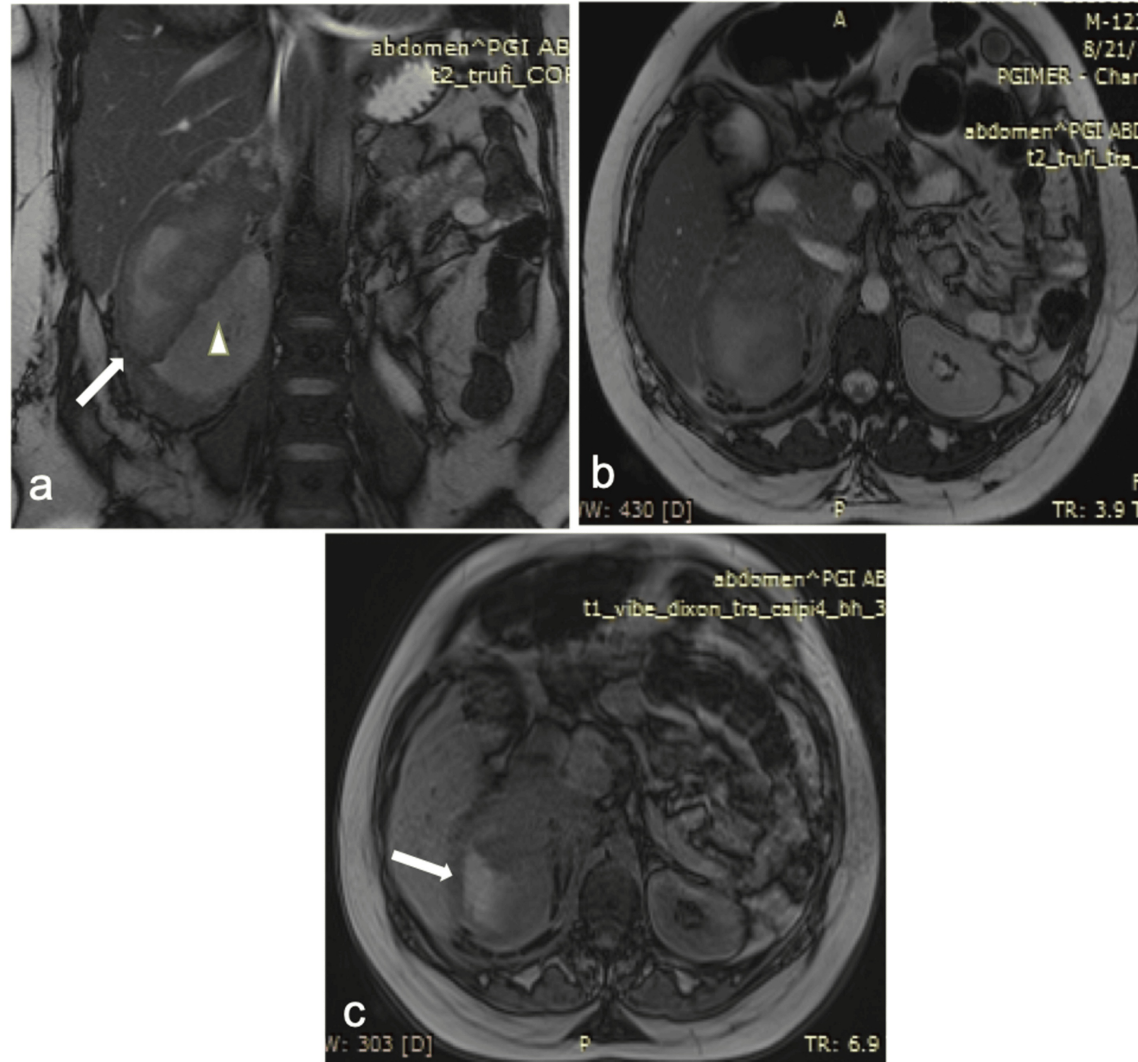
MRI findings. (a, b) Coronal and axial T2-weighted true fast imaging with steady-state free precession images show a heterogeneous, predominantly hyperintense lesion in the right suprarenal region (arrow). (c) Axial T1-weighted out-phase image showing hyperintense contents (arrow) within the mass lesion representing subacute hemorrhage. The right adrenal gland is not separately identified from the hematoma suggesting right adrenal hemorrhage. The right kidney is displaced inferomedially by the hematoma (arrowhead in a).

A 24-year-old primigravida at 38 weeks and 6 days of gestation presented with acute-onset, right-sided loin pain radiating to the shoulder, tenderness, and fever for one day. She denied a history of trauma and gastrointestinal or urinary complaints. She was febrile (99.6°F), had a pulse rate of 96 beats/minute, and blood pressure of 156/90 mmHg. The fetal status was reassuring. Her hemoglobin was 9.9 g/dL, total leukocyte count was 21.2 × 10^9^/L, platelet count was 285 × 10^9^/L, blood urea was 17.13 mmol/L, and creatinine was 0.12 mmol/L. Her liver function tests and coagulation profile were normal. An ultrasound revealed a heterogenous hyperechoic lesion measuring 13 × 7 cm with no vascularity in the hepatorenal region compressing the right kidney. Considering the possibility of an abscess, broad-spectrum antibiotics were initiated. Blood and urine cultures were sterile and serum procalcitonin was 0.02 ng/mL. Non-contrast MRI showed a mass (9.4 × 7.3 × 11 cm) in the right suprarenal region appearing iso to hyperintense on T1 and T2-weighted images, suggestive of subacute hemorrhage (Figure 1).

**Figure 2:**
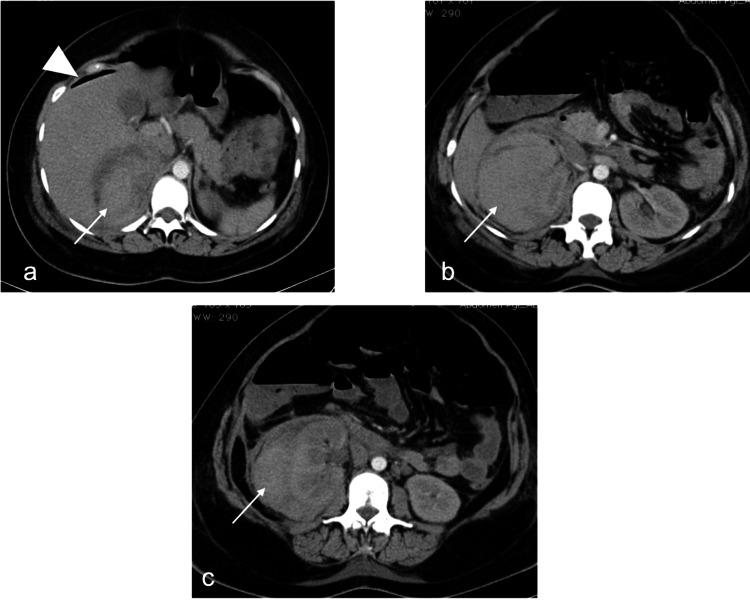
CT angiography findings. (a-c) Axial CT angiography images at the suprarenal and renal hilum level show heterogeneous enhancement with predominant hyperdense contents (arrow) in the right suprarenal space extending inferiorly into the perinephric space, suggesting right adrenal hemorrhage with hematoma. The right adrenal gland is not separately identified from the hematoma. A note is made of the pneumoperitoneum suggestive of the postoperative status (arrowhead in a).

As the hematoma did not progress in size and the patient was hemodynamically stable, conservative management was planned but termination of pregnancy was required due to pre-eclampsia. Due to concerns of a re-bleed during labor, a planned cesarean section was preferred. She delivered a healthy baby weighing 3.4 kg. Intraoperatively, a hematoma in relation to the right kidney was evident, for which expectant management was decided as it was non-pulsating and non-expanding. On postpartum day two, a contrast-enhanced CT (CECT) and CT angiography (CTA) confirmed a 13 × 10 × 6 cm hematoma in the right suprarenal and perinephric space and the absence of any pseudoaneurysm or bleeding vessel (Figure 2).

Ultrasound after six weeks showed a reduction in the size of hematoma and adrenal function was normal. After 18 months, the ultrasound showed kidneys of normal size and morphology.

Case 2

**Figure 3:**
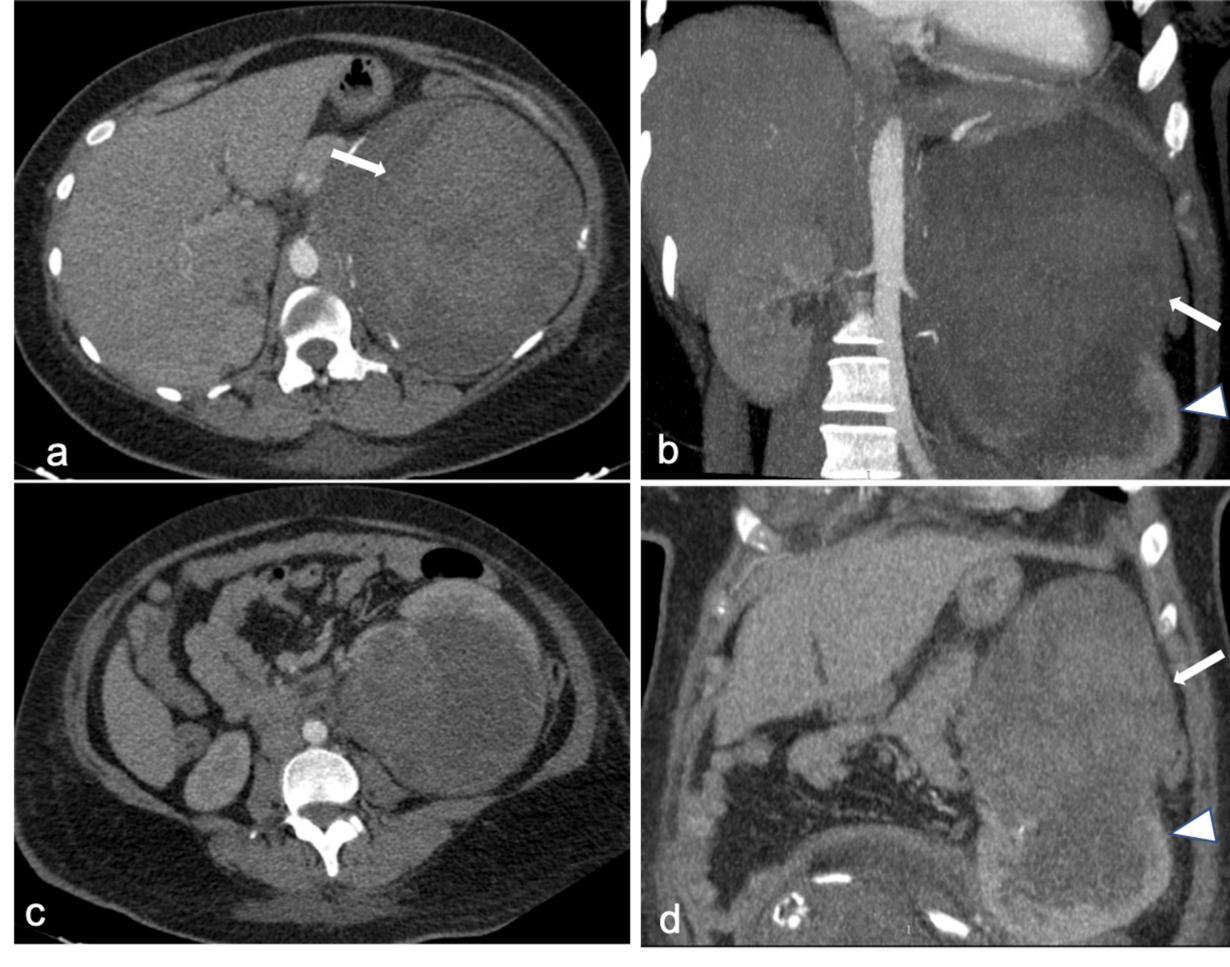
CT findings. (a, b) Axial and coronal arterial maximum intensity projection (MIP) images show heterogeneous enhancement with a predominant hyperdense component with minimal hypervascularity along the upper pole of the left kidney (arrow). (c, d) Axial and oblique coronal MIP images in the portal venous phase show similar findings with no obvious active bleeder or pseudoaneurysm. Normal enhancing lower pole of the left kidney can be noted (arrowhead).

A 25-year-old, gravida 4 with a previous normal vaginal delivery at term and two abortions, presented at 35 weeks and 2 days of gestation with chronic hypertension and superimposed pre-eclampsia with complaints of shortness of breath and swelling throughout the body for two days. On examination, she was afebrile, looked pale (hemoglobin 5.4 g/dL), had a pulse rate of 96 beats/minute, and blood pressure of 170/110 mmHg. Abdominal examination revealed a gravid uterus of 30 weeks with fetal demise. Her other blood investigations were as follows: total leukocyte count of 15,500 cells/mm^3^, platelet count of 393 × 10^3^/mm^3^, blood urea of 52 mg/dL, serum creatinine of 1.06 mg/dL, serum bilirubin of 0.5 mg/dL, aspartate transaminase of 172 IU/mL, alanine transaminase of 114 IU/mL, and alkaline phosphatase of 380 IU/mL. Ultrasound showed a large hyperechoic mass lesion in the left kidney, and fetal demise with no evidence of placental abruption. CECT and CTA showed a large hyperdense hematoma with an underlying hypodense mass lesion along the upper pole of the left kidney (Figure 3) without any pseudoaneurysm.

No macroscopic fat component was separately identified from the hematoma, possibly suggesting fat-poor angiomyolipoma with rupture/bleed. One week back, her hemoglobin was 9.2 g/dL. Considering the fall in hemoglobin from 9.2 g/dL to 5.4 g/dL and CT findings, the diagnosis of hemorrhage into the tumor was upheld. As there was no active bleeding or prominent arteries on CTA, labor was allowed with the provision of emergency embolization as a backup. She delivered a stillborn baby weighing 1.8 kg. Follow-up ultrasound after six weeks showed a 12 × 16 cm heterogeneously hyperechoic mass, suggestive of angiomyolipoma with minimal vascularity. At present, the patient is fine, and surgery for the renal tumor is planned.

Case 3

A 33-year-old, gravida 3, with two previous normal vaginal deliveries, presented at 33 weeks and 4 days gestation with twins with gestational hypertension and acute abdomen. On examination, the patient was pale (hemoglobin: 5.3 g/dL), had a pulse rate of 120 beats/minute, and blood pressure was 90/60 mmHg. Her abdomen was tense and overdistended, and fetal heart sounds were absent. Her leukocyte count was 17.1 × 10^9^/L, platelet count was 108 × 10^9^/L, blood urea was 19.9 mmol/L, and serum creatinine was 0.17 mmol/L, and international normalized ratio of 1.17. Ultrasound showed a large heterogenous lesion in the upper abdomen; the right kidney was not visualized separately. The obstetric scan revealed twins with no cardiac pulsations, adequate amniotic fluid, and normal placenta.

Along with resuscitation with fluids and blood components (packed red cells and fresh frozen plasma), an exploratory laparotomy was done. Both fetuses weighing 1.78 kg and 1.74 kg were delivered by hysterotomy. The mass was found to be a right-sided zone 2 hematoma limited to the right fascia of Gerota. There was a very high chance of hemodynamic compromise with further exploration. As the patient’s hemodynamics improved, the possibility of ongoing bleeding was low. Hence, expectant management of hematoma was decided. On postoperative day two, the patient had tachycardia, hypotension, and a drop in hemoglobin to 4 g/dL. An emergency exploratory laparotomy was done. Intraoperatively, there was active bleeding from the renal mass measuring 30 × 25 cm. A right-sided nephrectomy was done. The patient was discharged in stable condition. Histopathology revealed angiomyolipoma.

## Discussion

Retroperitoneal hemorrhage caused severe maternal morbidity and fetal demise in two of our patients. Although ultrasound is the first imaging modality used to evaluate abdominal pain in pregnancy [2], it may not be conclusive for retroperitoneal pathologies in the late third trimester due to gravid uterus. MRI is the most accurate imaging study for adrenal hemorrhage [2]. In Case 1, the mass lesion in relation to the kidney was initially thought to be an abscess. MRI helps in better characterization of the mass as a hematoma [2]. As the hemodynamic condition of the first patient was stable, conservative management [1] was planned. Emergency adrenalectomy may be required if the hemorrhage results in hemodynamic instability [1]. Once conservative management for hematoma was decided, the next challenge was to decide the mode of delivery because she had pre-eclampsia too. The possibility of re-bleeding due to increased intra-abdominal pressures in labor is always a concern. In a review of 11 patients who had an adrenal hemorrhage in the antenatal period, two had fetal demise, five underwent cesarean section due to maternal-fetal compromise (four) or risk of repeat hemorrhage during labor (one), four patients with alive fetuses who had hemorrhage remote from term underwent labor resulting in vaginal delivery (three), or emergency cesarean section for the obstetric indication (one) at term [1,2,4]. Labor may be allowed if delivery is spaced by at least a few weeks from the occurrence of adrenal hemorrhage [1,6]. When hemorrhage occurs close to delivery, cesarean section is opted for [2]. Hence, we decided on a cesarean section after counseling the patient and her family as the hemorrhage was recent.

Renal angiomyolipomas mostly remain asymptomatic [5]. Hemorrhage into the tumor is a rare but dreaded complication. An increase in size during pregnancy [9,10], increased intra-abdominal pressure, and changing hemodynamics predispose these tumors to rupture [7]. Some studies have even suggested termination of pregnancy if diagnosed early [10]. Prophylactic interventions ranging from SAE and nephron-sparing surgery to total nephrectomy have been described, especially where emergency care is not available [7-9,11]. Earlier, it was widely accepted that tumors measuring >4 cm should be treated because they are more likely to bleed (>50%) [12]. Recently, this cut-off value as a predictor of hemorrhage has been challenged [13]. In patients who present with hemorrhagic shock and fetal demise without any prior diagnosis, management becomes challenging. In Cases 2 and 3, the initial diagnosis was concealed placental abruption until an ultrasound showed a normal placenta and a mass lying outside the uterus. Although obstetric causes are always considered first, it is important to entertain non-obstetric differentials early in the course of the investigation. An ultrasound in an emergency can help clinch the diagnosis of alternate pathology. Once the obstetric cause is not obvious on ultrasound, the umbrella of imaging should be expanded to include the upper abdomen and kidneys.

In the absence of standard guidelines for obstetric management in angiomyolipomas, it should be individualized. As Case 2 had stable vitals, the trial of labor was given with the provision of embolization kept ready. On the other hand, Case 3 presented with hemorrhagic shock. Following resuscitation with intravenous fluids and blood transfusion, the patient’s hemodynamic parameters reverted to normal. Cesarean section might have also contributed to improvement due to better venous return. As the evacuation of the hematoma would release the tamponade effect and lead to nephrectomy, conservative management was planned, but she had to undergo an emergency nephrectomy later. Embolization with [9] or without [14] emergency nephrectomy for smaller tumors in the mid-trimester may allow continuation of pregnancy to term. For tumors with mild bleeding, close observation or arterial embolization can be done [15], but only in places where facilities for emergency surgery are available.

## Conclusions

Retroperitoneal hemorrhage is rarely suspected as a cause of pain and/or shock in pregnancy. Once the common obstetric causes are ruled out, non-obstetric causes should be explored early in the course of the diagnostic workup. Timely detection of retroperitoneal hemorrhage prevents catastrophic outcomes. Ultrasound can detect or raise suspicion and guide management.

Due to its rarity, the management of retroperitoneal hemorrhage in pregnancy due to adrenal or renal causes requires an individualized approach. Immediate recruitment of a multidisciplinary team comprising Obstetrics, Urology, Transfusion Medicine, Anesthesia, Neonatology, Radiodiagnosis, and Intervention Radiology (for embolization) holds the key to favorable outcomes. The involvement of the patient and her family in making decisions is equally important.
